# Spices and Essential Oils in Food Preservation: From Natural Additives to Active Packaging Systems

**DOI:** 10.1007/s11130-026-01519-8

**Published:** 2026-05-26

**Authors:** Olena Stabnikova, Viktor Stabnikov, Octavio Paredes-López

**Affiliations:** 1 Advanced Research Laboratory, National University of Food Technologies, Kyiv, Ukraine; 2 Department of Biotechnology and Microbiology, National University of Food Technologies, Kyiv, Ukraine; 3 Departamento de Biotecnología y Bioquímica, Centro de Investigación y de Estudios Avanzados del Instituto Politécnico Nacional, Irapuato, 36821 Gto México

**Keywords:** Spices, Essential oils, Antimicrobial activity, Antioxidant activity, Food preservation, Active packaging

## Abstract

This review provides a systematic analysis of ten spices representing the largest share of global production, with a particular focus on their antimicrobial and antioxidant properties and their role in food preservation. These bioactive properties are of special importance for ensuring food safety, maintaining product quality, and extending shelf life. The review analyzes current approaches to the application of spices in the food industry, both in their natural form and as essential oils. The effectiveness of essential oils is analyzed in various applications, including their use as food additives, surface treatment agents, and components of edible coatings. Advanced delivery systems, such as micro- and nanoencapsulation and nanoemulsion-based formulations, are discussed as promising strategies to enhance stability, bioavailability, and controlled release. Special attention is given to the incorporation of spices into the development of active packaging systems. Additionally, the review addresses current challenges and limitations associated with the use of spices and their derivatives in processed food production and highlights future perspectives for their rational and sustainable use.

## Introduction

Spices are natural plant substances used to enhance the flavor of foods. According to the United States Department of Agriculture, spices and herbs are plant materials that impart flavor to dishes [1]. While spices can be derived from roots, stems, leaves, bark, flowers, fruits, or seeds, herbs are typically non-woody plants. The term “spice” originates from the Late Latin word *species*, meaning aromatic substances. These ingredients possess characteristic aromas and are typically added in small quantities to enhance flavor and, in some cases, retard spoilage. They contain a variety of bioactive compounds, including essential oils, polyphenols, glycosides, and tannins, many of which exhibit anti-inflammatory, antioxidant, antimicrobial, and anticancer activities. Spices are usually dried and sometimes ground to preserve and enhance their sensory and functional properties. While dried spices have a long shelf life, fresh forms, such as ginger, often have a more intense flavor but tend to be more expensive and more perishable.

Because spices are poorly preserved, they are rarely recovered in archaeological contexts. Nevertheless, evidence suggests that spices were used in food as early as prehistoric times. In the western Baltic region, garlic mustard seeds were identified in 6,000-year-old ceramic cooking pots [2], while ginger starch grains recovered from human dental calculus at the sites of Harappa (Pakistan) and Farmana (India) date to approximately 4,000–4,500 years ago [3]. In Southeast Asia, residues of turmeric, ginger, cloves, and cinnamon were detected on ancient stone tools from the Óc Eo site (Vietnam), dating back about 2,000 years [4].

Since ancient times, spices have played an important role not only in food but also in medicine, ritual practices, and trade, contributing to intercultural exchange and the development of early trade networks. The spice trade, originating more than 4,000 years ago in regions such as India and Southeast Asia, was historically mediated by Middle Eastern traders and later by European trade centers, including Venice, which controlled key trade routes between East and West [5]. Spices such as black pepper, cinnamon, cloves, and ginger were highly valued in ancient Greece and Rome and were considered luxury commodities.

**Fig. 1:**
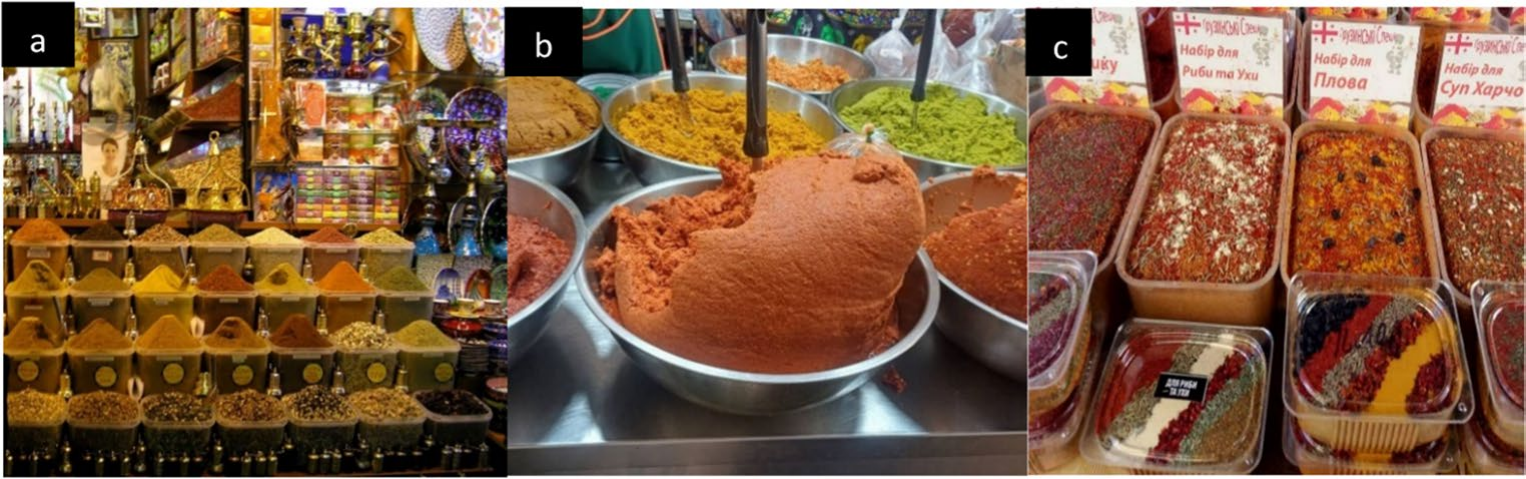
Spice markets in Istanbul, Turkey (**a**), in Samut Prakan, Thailand (**b**), and Kyiv, Ukraine (**c**) (Photos: a, c by O. Stabnikova; b by Bilal Khalid, used with permission)

The high economic value of spices stimulated long-distance trade and contributed to the development of global maritime exploration routes. Over time, the discovery of new continents introduced additional aromatic plants, such as chili peppers, vanilla, and cacao, into global trade networks. Today, spices are widely available and used worldwide in both culinary and industrial applications (Fig. 1).

Advances in global trade, transportation, and preservation have made spices more readily available, while their culinary, medicinal, and cultural uses remain important. In the modern world, Asia (including countries such as India, Indonesia, Sri Lanka, Malaysia, China, and Vietnam), South America (Brazil, Peru, and Colombia), and Africa (notably Madagascar, Tanzania, and Ethiopia) are the main producers of spices [6].

**Fig. 2:**
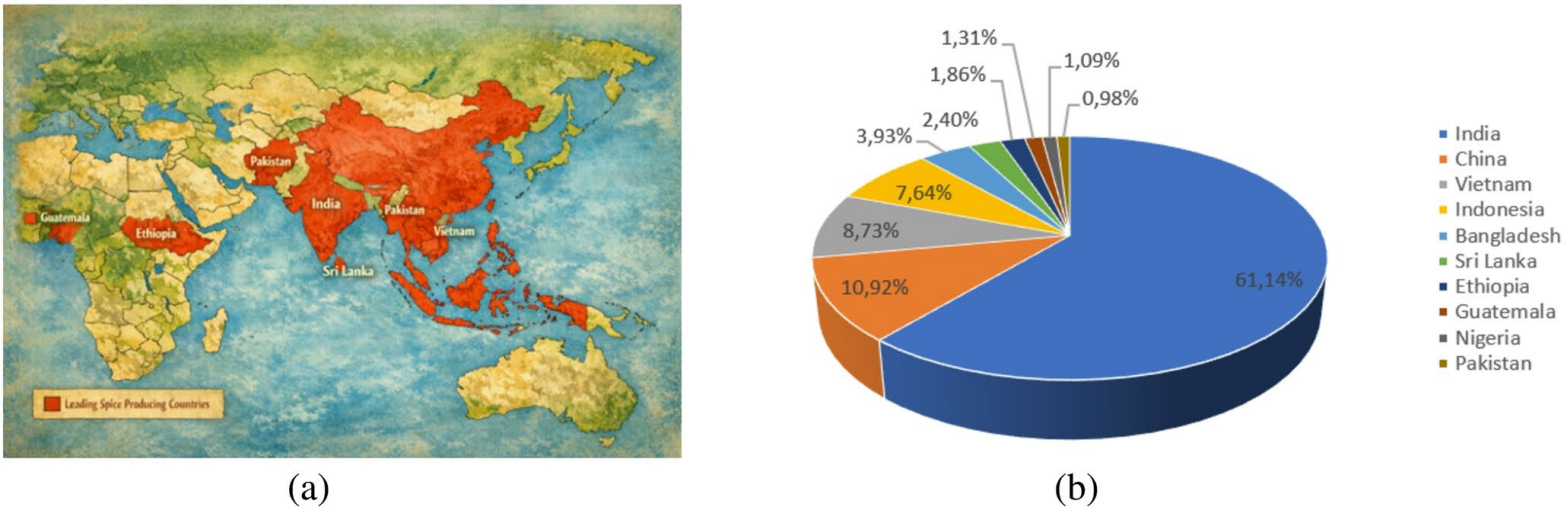
Major spices (**a**) and percentage share of the top 10 spice-producing countries in total global spice production (**b**)

In 2023, the global spices market amounted to around USD 18 billion and is expected to grow steadily, reaching approximately USD 24 billion by 2030 at a CAGR of 5.1%. This growth is driven not only by the widespread culinary use of spices but also by their expanding application in the wellness, pharmaceutical, and cosmetic sectors, which has led to increased worldwide demand. According to recent industry data reported by uFoodin [6], India is the leading global producer of spices, with an annual production of approximately 2.8 million metric tons, followed by China and Vietnam. Ten countries that are widely recognized as major spice producers are shown in Table 1; Fig. 2.

**Table 1:** Top countries for spice production (adapted from [6])

Rank	Country	Production, metric tons per year	Major spices
1	India	2,800,000	Turmeric, chili, cumin, and cardamom
2	China	500,000	Chili, ginger, cinnamon and star anise
3	Vietnam	400,000	Black pepper, cinnamon and star anise
4	Indonesia	350,000	Nutmeg, clove, and vanilla
5	Bangladesh	180,000	Turmeric and ginger
6	Sri Lanka	110,000	Cinnamon
7	Ethiopia	85,000	Chili, ginger, and black cumin
8	Guatemala	60,000	Cardamom
9	Nigeria	50,000	Chili, ginger, and turmeric
10	Pakistan	45,000	Chili, cumin, and fennel

There short characteristics of major spices produced and consumed worldwide according to Table 1.

Black pepper (*Piper nigrum*) is among the most widely used spices in the world, obtained from dried unripe berries and valued for its sharp flavor and aroma. Its main bioactive compound, the alkaloid piperine (5.3–9.2%), exhibits antioxidant, anti-inflammatory, and antimicrobial properties [7, 8]. It is widely used in meat and vegetable dishes, sauces, marinades, soups, and curries, imparting a characteristic pungent flavor.

Black cumin (*Nigella sativa*) is obtained from seeds and is valued for its distinctive aroma and slightly bitter, peppery taste. Its main bioactive compounds, including thymoquinone and *p*-cymene, exhibit antioxidant, anti-inflammatory, and antimicrobial properties. The seeds are commonly used in Middle Eastern and Asian cuisines, including bread, pastries, pickles, and curries, and are also a component of spice blends such as panch phoran [9].

Cardamom (*Elettaria cardamomum*) consists of seeds enclosed in dried capsules and is highly valued for its rich aroma, primarily due to α-terpinyl acetate and 1,8-cineole [10]. It is widely used in both sweet and savory dishes, as well as in beverages such as tea and coffee, and in traditional spice blends [11].

Chili peppers and paprika are derived from dried fruits of *Capsicum annuum*. The pungency of chili peppers is mainly attributed to capsaicin (up to 1–2% dry weight) [12], whereas paprika contains little or no capsaicin and is characterized by volatile compounds such as terpenoids and esters. Their flavor profiles depend on variety and processing conditions. Chili peppers are widely used in spicy dishes, while paprika is used to provide color, mild flavor, and aroma [13, 14].

Cinnamon is obtained from the dried inner bark of trees of the genus *Cinnamomum*. *Cinnamomum verum* (“true” cinnamon) is distinguished from cassia types, which are more widely marketed. The main compound responsible for its aroma is cinnamaldehyde [15]. Cinnamon is used in both sweet foods and beverages, as well as in savory dishes in Middle Eastern and Asian cuisines [16, 17].

Cumin (*Cuminum cyminum*) is obtained from seeds and is characterized by compounds such as cuminaldehyde and *p*-cymene [18]. It is widely used as a seasoning in meat, legume, and rice dishes and is a key component of many spice blends across different culinary traditions [19].

Cloves, the dried flower buds of *Syzygium aromaticum*, owe their characteristic aroma and bioactivity to eugenol [20]. They are widely used in spice blends and to flavor marinades, sauces, and meat dishes.

Fennel (*Foeniculum vulgare*) fruits, commonly referred to as seeds, are characterized by a licorice-like flavor due to anethole (3–5%) [21]. They are used in both sweet and savory foods, as well as in beverages and spice mixtures.

Ginger (*Zingiber officinale*), obtained from dried rhizomes, contains compounds such as zingiberene that contribute to its characteristic pungent flavor and bioactivity [22]. It is widely used in a variety of culinary traditions, including savory dishes and beverages.

Nutmeg (*Myristica fragrans*) is derived from seeds, while mace is obtained from the aril. Its main bioactive compounds include myristicin and elemicin [23]. It is used in both sweet and savory foods and contributes to flavor as well as antimicrobial effects [24].

Turmeric (*Curcuma longa*) is obtained from rhizomes and is characterized by its bright yellow color and the presence of curcumin [25]. It is widely used in cooking and traditional preparations, particularly in Asian cuisines.

Star anise (*Illicium verum*) is a fruit rich in trans-anethole, which determines its characteristic aroma [26]. It is used to flavor both sweet and savory dishes and is a component of traditional spice blends.

### Characteristic Compounds of Spices and Their Essential Oils

Spices are widely used to add flavor and aroma to foods. In addition, they can enrich foods with minerals, vitamins, and antioxidants, which improve food quality, protect against oxidation, and help extend shelf life. Although spices contain a wide range of minerals and vitamins and serve as important raw materials for the production of essential oils. Bioactive compounds present in spices and their essential oils can be broadly grouped into several major chemical classes, including phenolic compounds (such as phenolic acids, flavonoids, and phenylpropanoids, including eugenol, cinnamaldehyde, estragole, and anethole), terpenoids (such as monoterpenes, including pinene, myrcene, limonene, terpinene, and p-cymene, and sesquiterpenes, including caryophyllene, humulene, and farnesol), quinones (such as thymoquinone), and organic acids.

The antimicrobial activity of essential oil components depends largely on their hydrophobicity, allowing them to pass through the lipid-rich cell membranes of bacteria, yeasts, and molds. Due to their lipophilic nature, many terpenoids and phenylpropanoids can penetrate the phospholipid bilayer, disrupt membrane structure, thereby increasing membrane permeability and causing leakage of intracellular constituents such as ATP, ions, and nucleic acids, leading to cell death [27]. In food matrices, the effectiveness of essential oils is strongly influenced by their partitioning between the aqueous and lipid phases, as well as their interactions with proteins, fats, and carbohydrates, which can reduce the bioavailability of active compounds and alter their antimicrobial efficacy. Since essential oils are predominantly lipophilic, their antimicrobial activity largely depends on the concentration of active compounds present in the aqueous phase, where most spoilage and pathogenic microorganisms are located [28].

Phenolic compounds are widely associated with antioxidant activity due to their ability to donate hydrogen atoms or electrons and stabilize free radicals. Terpenoids, as major constituents of essential oils, are primarily responsible for antimicrobial activity through interactions with microbial cell membranes, leading to disruption of cellular integrity. Quinones and organic acids also contribute to antimicrobial effects through redox activity, pH reduction, and interference with microbial metabolism. Table 2 shows the characteristic compounds of spice (minerals and vitamins) and their essential oil compositions.

**Table 2:** Characteristic compounds of spices and their essential oil

Spice	Valuable compounds in raw materials		Major components (%) of the corresponding essential oil
	Minerals, mg/100 g [29]	Vitamins, mg/100 g [29]	
Black cumin	Phosphorous 499; potassium, 1790; sodium, 168; iron, 66.4	Niacin, 4.58; thiamine, 0.63; pyridoxine, 0.44; α-tocopherol, 3.33	Thymoquinone, 30–48; p-cymene, 7–15; trans-anethole, 1–4; sequiterpene longifolene, 1–8 [30]
Black pepper	Phosphorous 158; potassium, 1330; calcium, 443; iron, 9.71	Niacin, 1.14; riboflavin, 0.18; thiamine, 0.11; β-carotene, 0.31	β-caryophyllene, 25.38; limonene, 15.64; sabinene, 13.63; 3-carene, 9.34; β-pinene; 7.27; α-pinene, 4.25 [31]
Cardamom	Phosphorous, 178; potassium, 1119; calcium, 383; magnesium, 229; iron, 14	Niacin, 1.1; thiamine, 0.19; riboflavin, 0.18; pyridoxine, 0.23; vitamin C, 21	1.8-cineole, 55.4; α-terpinyl acetate, 28.6; 4-terpineol, 4.3; α-terpineol; limonene, 2.5; sabinene, 1.5 [32]
Cinnamon	Phosphorous, 64; potassium, 431; calcium, 1000; iron, 8.3; magnesium, 60	Niacin, 1.33; ascorbic acid, 3.8; pantothenic acid, 0.36; pyridoxine, 0.158	(E)-cinnamaldehyde, 71.50; linalool, 7.00; β-caryophyllene, 6.40; eucalyptol, 5.40; eugenol, 4.60 [33]
Chili	Phosphorous, 300; iron, 17.3; magnesium, 149; potassium, 1950; sodium, 2870	Niacin, 11.6; riboflavin, 0.94; pyridoxine, 2.09; vitamin C, 0.7; pantothenic acid, 0.88, vitamin A, 5.18	Palmitic acid, 10.1; furfural, 5.6; pentadecanal, 5.3;1-nonadecene, 3.5; (E)-geranylacetone, 1.1; linoleic acid, 1.0; linolenic acid, 0.9 [34]
Clove	Phosphorous, 1020; calcium, 632; potassium, 1102; magnesium, 259; iron, 11.8	Niacin, 1.56; riboflavin, 0.22; pantothenic acid, 0,509; thiamine, 0.158; ascorbic acid, 0.2	Eugenol, 82.16; acetyleugenol, 16.25; caryophyllene, 0.79 [35]
Cumin	Phosphorous, 499; potassium, 1790; magnesium, 366; iron, 66.4	Niacin, 4.58; thiamine, 0.63; ascorbic acid, 7.7; α-tocopherol, 3.3; β-carotene, 0.762; vitamin A, 0.381	Limonene, 36.77; carvone, 57.94; α-pinene, 1.58; trans-dihydrocarvone, 1.66 [36]
Fennel	Phosphorous, 487; potassium, 1694; calcium, 1200; sodium, 88; iron, 18.5	Niacin, 6.05; thiamine, 0.408; riboflavin, 0.353; pyridoxine, 0.47; vitamin A, 0.041; vitamin C, 21	Estragole, 66.1–85.2; fenchone, 5.2–23.1; limonene, 4.3–10.3 [37]
Ginger	Phosphorous, 168; magnesium, 214; calcium, 114; potassium, 1329; iron, 19.8	Niacin, 9.62; riboflavin, 0.17; pantothenic acid, 0.477; pyridoxine, 0.626; vitamin C, 0.7	α-zingiberene, 22.18; β-sesquiphellandrenene, 11.05; 1.8-cineol, 6; geranial, 5.13; β-bisabolene, 4.96 [38]
Nutmeg	Phosphorous, 213; potassium, 350; calcium, 184; iron, 3.04; magnesium,183	Niacin, 1.3; thiamin, 0.346; pyridoxine, 0.16; vitamin C, 3	Sabinene, 21.71; α-pinene, 15.81; myristicin, 13.39%; β-pinene, 12.70 [39]
Paprika	Phosphorous, 314; potassium, 2280; magnesium, 178; calcium, 229; sodium, 68; iron, 21.1	Niacin, 10.1; riboflavin, 1.23; pyridoxine, 2.14; pantothenic acid, 2.51; lutein+zeaxanthin, 18.9; vitamin A, 14.79; α-tocopherol, 29.1; β-carotene, 26.2	α‑curcumene,16.0; β‑sesquiphellandrene, 14.7; β-caryophyllene, 13.0; β-bisabolene, 10.5; limonene, 8.6; linalool, 6.6; α-copaene 4.1 [14]
Turmeric	Phosphorous, 299; potassium, 2080; magnesium, 208; calcium, 168; iron, 55	Niacin, 1.35; pantothenic acid, 0.542; riboflavin, 0.15; pyridoxine, 0.107; ascorbic acid, 0.7	Ar-turmerone (30.3%), α-turmerone (26.5%), β–turmerone (19.1%) [40]

In addition to essential oils, spices are used to produce oleoresins - concentrated, multicomponent extracts obtained by solvent extraction followed by solvent removal, yielding an oily liquid or powder that preserves the characteristic aroma and flavor of the original spice. Oleoresins represent an effective alternative to ground spices due to their improved storage stability and longer shelf life. Production of macerates involves the extraction of flavor and bioactive compounds from spices into edible oils through prolonged contact, resulting in aromatic preparations widely used in food applications [41].

### Spices for Functional Food Production

A healthy diet involves consuming nutrient-dense foods while limiting added sugar, saturated fat, and sodium. Nevertheless, excessive intake of these components remains a global challenge [42]. Because palatability strongly influences consumer acceptance, products reformulated to contain lower levels of sugar, fat, and sodium must maintain an appealing flavor. Incorporating herbs and spices is a promising strategy to enhance flavor while reducing these components [43, 44], and many national and international dietary guidelines recommend their use to support healthy eating. Consumer surveys also show that products containing natural spices are highly rated as more aromatic, flavorful, and visually appealing, with high overall acceptance [45, 46].

Clinical trials and sensory studies have shown that herbs and spices can effectively replace added sugars, saturated fat, and sodium, although achieving widespread dietary change requires public education. For example, a randomized clinical trial conducted from 2012 to 2014 found that a 20-week behavioral intervention teaching adults to use herbs and spices for flavor significantly reduced 24-h urinary sodium excretion by nearly 1,000 mg/day compared with a self-directed control group [47].

Reducing sugar intake is an important public health objective; however, many consumers are reluctant to use low-calorie sweeteners such as aspartame, stevia, and sucralose. These substitutes have several limitations: they may maintain a preference for sweet taste, affect appetite regulation and gut microbiota, provide no nutritional value, lead to compensatory eating, and potentially exert adverse effects on human health [48, 49]. Moreover, reducing sugar levels may partially limit Maillard reaction-derived flavor and aroma formation, depending on the food matrix and processing conditions [50]. For these reasons, experts increasingly recommend the use of natural flavorings, particularly culinary spices, to reduce sugar content while maintaining the palatability of foods.

Clinical trials have shown that adding culinary spices to reduced-sugar foods can preserve their hedonic appeal and maintain consumption. In studies with adults aged 18–65, sugar was reduced by up to 37% in apple crisp, and when spices were added, overall liking and consumption remained comparable to the full-sugar version on a 9-point hedonic scale. These results suggest that incorporating spices into reduced-sugar foods is an effective strategy to make lower-sugar options acceptable while maintaining taste, supporting healthier eating patterns in free-living populations [51].

In a randomized sensory clinical trial, reducing fat content by approximately 50% led to a significant decrease in overall meal liking compared with the full-fat condition (mean score 6.29 vs. 7.05). However, when herbs and spices were added to the reduced-fat foods, overall liking was restored to a level comparable with full-fat meals (6.98 vs. 7.05). The addition of herbs and spices eliminated differences in liking for reduced-fat meatloaf and vegetables relative to full-fat versions and significantly improved the acceptability of reduced-fat pasta. These results demonstrate that culinary spices can effectively compensate for flavor loss associated with fat reduction and support the development of lower-fat foods without compromising palatability [52].

In another study, it was shown that adding a mixture of herbs and spices (including garlic, onion, black pepper, paprika, and cinnamon) to foods with reduced levels of total fat (42% in a breakfast meal and 59% in a lunch meal), saturated fat (50% and 68%, respectively), and energy (15% and 27%, respectively) resulted in similar ratings compared to full-fat meals when assessed using a 9-point hedonic scale: breakfast: 6.82 vs. 6.84; lunch: 6.71 vs. 6.94, whereas reduced-fat meals without added spices scored lower (breakfast: 6.50; lunch: 6.35). This demonstrates that herbs and spices can restore flavor in lower-fat foods while substantially reducing caloric content [53].

Petersen et al. [42] studied the use of herbs and spices to improve the flavor of 10 commonly consumed foods, including meatloaf, chicken pot pie, macaroni and cheese, and brownies, reformulated to reduce saturated fat, sodium, and added sugars. For example, in the reformulated macaroni and cheese recipe, the researchers replaced salted butter with unsalted butter (reducing its amount by 75%), switched to skim milk and reduced‑fat cheese, removed extra salt, and added spices such as onion powder, garlic powder, ground mustard seed, paprika, and cayenne to enhance flavor. The addition of these spices improved sensory acceptance in blind taste tests, with seven out of ten reformulated dishes rated equal to or higher than the original versions. If 25–100% of consumers adopted these reformulated foods, estimated daily intake of saturated fat decreased by 2.9–11.4%, sodium by 3.2–11.5%, and added sugars by 0.5–2.7%, demonstrating that herbs and spices can enhance flavor while supporting healthier dietary choices. Culinary spices have been shown to enhance liking and perceived flavor intensity of protein-rich foods in older adults. Although spice addition did not significantly increase total protein intake, it improved sensory acceptance of both meat- and plant-based meals, particularly vegetarian dishes, highlighting the potential of spices to support dietary compliance in aging populations [54].

Together, these findings highlight culinary spices as an effective, natural strategy to support reductions in over consumed dietary components, such sodium, added sugars, and fat, while maintaining food enjoyment and consumer acceptance. At the same time, spices in the form of powders, fresh, extracts, essential oils, or oleoresins can be used as flavor-, color-, and aroma-enhancing agents, as well as for food preservation, due to their antioxidative and antimicrobial properties.

#### Application of Spices in Functional Bakery Products

The direct addition of clove or cumin essential oils (EO) (1% w/w) to wheat-based products significantly improved their oxidative stability and shelf life. Over 60 days of storage, sweet biscuits with clove EO showed only ≈ 5.2% increase in free fatty acids and ≈ 1.3% rise in peroxide value, while cumin EO salt biscuits had ≈ 6.1% and ≈ 1.5% increases, respectively, compared with ≈ 106% and ≈ 18.5% in the control. These findings indicate that essential oils can effectively reduce lipid oxidation and rancidity, offering a natural preservative and flavor-enhancing strategy [55]. The addition of coriander essential oil at concentrations of 0.05–0.1% improved the oxidative stability and shelf life of cakes by reducing lipid oxidation and inhibiting fungal growth over 60 days of storage. However, a concentration of 0.15% negatively affected sensory quality, whereas 0.05% maintained the cake’s acceptability [56].

#### Application of Spices in Functional Dairy Products

A modern trend in dairy production involves the wide use of spices [57]. The global spicy dairy market was valued at USD 680.95 million in 2023 and is expected to grow at a CAGR of 6.2% from 2024 to 2033. Spicy dairy products such as cheese, butter, ice cream, cottage cheese, and yogurt combine a creamy texture with the rich flavor of spices and offer unique aromas. Dairy products serve as the foundation for these aromas and flavors, driving broad consumer demand across various segments of the population.

#### Yogurt

Adding 1.5% (w/v) cinnamon powder to yogurt significantly increased the total polyphenol content and antioxidant activity [58]. Although only about 34.7% of the polyphenols from the added cinnamon were initially present in a free form due to interactions with milk proteins, simulated gastro-pancreatic digestion released these compounds, increasing their bioaccessibility to approximately 60%. Consequently, cinnamon-enriched yogurt exhibited 2–3-fold higher antioxidant activity than plain yogurt, while cinnamaldehyde remained relatively stable during digestion. Based on these findings, the authors suggested that cinnamon-fortified yogurt may contribute to protecting the gastrointestinal tract against free radical–induced damage.

Incorporation of spices in sour milk paste, which contains up to 10% sugar and is commonly used in desserts such as curd products and cream, demonstrated clear functional and technological benefits. A mixture of aromatic spices (sweet pepper, ginger, cardamom, and fenugreek in a ratio of 1:1:0.8:1.2) added at 0.27–1.1% (w/w), significantly increased the content of minerals and biologically active substances, particularly vitamins and phenolic compounds (up to 223.4 mg per 100 g of sour milk paste). This spice composition exhibited strong antioxidant and antimicrobial activities, inhibited lipid oxidation, improved storage stability, and enhanced sensory properties, supporting the development of sugar-free, clean-label dairy desserts with increased nutritional value [59].

Probiotic yogurts supplemented with 0.5% (w/v) spice oleoresins from cardamom, nutmeg, or cinnamon, obtained high overall score 6.22, 5.39, and 4.89, respectively, compared with 5.22 in the control. The addition of oleoresins also supported probiotic viability, with *Lactobacillus acidophilus* counts on day 28 of storage reaching 5.7 × 10⁷, 1.7 × 10⁸, and 1.6 × 10⁸ CFU/g for cardamom-, nutmeg-, and cinnamon-supplemented yogurts, respectively, compared with 3.5 × 10⁷ CFU/g in the control. Moreover, spice-supplemented yogurts demonstrated enhanced antioxidant activity in the DPPH assay (25.3%, 16.0%, and 35.0% vs. 11% in control). These findings indicate that spice oleoresins can simultaneously improve both the functional and sensory properties of probiotic yogurt [60].

#### Cheese

Cheese is not only a valuable and nutritious dairy product but also an important element of the culinary heritage of many countries. The use of spices in cheese making is widespread and they may be incorporated into the curd or applied to the cheese surface. Spices impart distinctive flavors and contribute to product stability and extended shelf life due to their antioxidant and antimicrobial properties. Examples of cheese with spices include Cheddar, Monterey Jack with chili (*Capsicum annuum*), Bouletted’Avesnes with paprika (*Capsicum frutescens*), Gouda with black pepper (*Piper nigrum*), Gouda, Leidsekaas with cumin (*Cuminun cyminum*), Moutardier with mustard seeds (*Brassica sinapis alba*) and others [61].

Usually, the amount of spice added to cheese curd ranges from 0.1 to 3% (w/w) relative to the milk used. Tarakçı and Deveci [62] evaluated the effects of spice incorporation at approximately 1% (w/w) on the chemical, biochemical, textural, and sensory properties of White cheese during ripening and reported significant effects on proteolysis, lipolysis, texture, and flavor development. Spiced cheeses, particularly those containing black cumin and red pepper, exhibited higher overall sensory scores than the control due to enhanced aroma and flavor intensity without adverse effects on texture. In a related study, White cheeses supplemented with sweet red (capia) pepper at 0.1–0.4% (w/w) and ripened for three months at 6 ± 2 °C showed improved sensory acceptance, with the 0.4% addition level being the most preferred, while changes in color and sensory attributes were not statistically significant [63].

Supplementation of Domiati soft cheese made from pasteurized milk artificially inoculated with a mixture of foodborne pathogens (*Staphylococcus aureus* ATCC 6538, *Escherichia coli* ATCC 8739, *Listeria monocytogenes* Scott A and *Salmonella enteritidis* PT4; ~4 log₁₀ CFU/mL) with black cumin seed oil (0.1–0.2%, w/w) showed significant antimicrobial activity, reducing pathogen counts by 1.3–1.5 log₁₀ CFU/g after 21 days of refrigerated storage. The oil exhibited particularly strong inhibitory effects against *E. coli* and *S. enteritidis*. In addition to improved microbial safety and extended refrigerated shelf life, black cumin seed oil supplementation helped maintain acceptable physicochemical and sensory properties of the cheese [64].

The addition of Sichuan pepper (*Zanthoxylum bungeanum*) and cumin (*Cuminum cyminum*) at 0.1 g/100 g of goat milk had no detrimental effect on the chemical composition of Camembert cheese, with moisture, protein, fat, and ash remaining comparable to the control during 40 days of ripening. In contrast, spices substantially enhanced antioxidant properties: total phenolic content increased 1.6–2.3 times, reaching 94.5 mg GAE/g in cumin cheese and 118.8 mg GAE/g in Sichuan pepper cheese versus 82.3 mg GAE/g in the control, while DPPH activity rose from 60.4% in the control to 73.5% and 87.8%, respectively. Sensory evaluation also improved, with spiced cheeses scoring higher for flavor, aroma, and overall acceptability, Sichuan pepper–containing samples being the most preferred, indicating substantial enhancement of both functional and sensory quality [65].

#### Butter

The addition of natural spice essences (w/w), cardamom (*Elettaria cardamomum*) at 0.25%, cinnamon (*Cinnamomum zeylanicum*) at 0.15%, and ginger (*Zingiber officinale*) at 0.20%, to unsalted butter effectively reduced rancidity development and improved keeping quality during five weeks of storage at 4 °C. Peroxide values, expressed as mEq O/kg, were 0.47 in butter containing cardamom or cinnamon and 0.54 in butter containing ginger, compared with approximately 3.8 in the untreated control. Moreover, the incorporation of spice essences significantly reduced free fatty acid content and lipolytic bacterial counts throughout storage, reflecting enhanced oxidative and microbiological stability [66].

#### Ice Cream

Ice cream is a popular dessert consumed by both children and adults. Although it provides essential nutrients derived from milk, it is generally deficient in biologically active compounds, particularly natural antioxidants such as vitamins and polyphenols. The incorporation of spices into ice cream is primarily intended to enhance aroma and flavor. Commonly used spices include cinnamon, cardamom, cloves, vanilla, ginger, nutmeg, and even black pepper. Spices may be incorporated in the form of powders [67–71], oleoresins [70, 72], or essential oils [73], either blended into the ice cream mix or added during processing to create distinctive dessert products.

Beyond their aromatic role, spices contribute to increased flavor complexity and serve as natural sources of antioxidants and other biologically active compounds. For example, low-fat ice cream supplemented with 0.1% of a black pepper–cinnamon powder mixture (1:3.75) exhibited significantly higher total phenolic content and antioxidant activity, expressed as mg Trolox equivalents (TE)/100 g sample, compared with the control (295.77 vs. 95.47 and 129.40 vs. 73.99, respectively), while sensory rating scores remained high [68].

The optimal level of spice addition is typically determined on an individual basis, as moderate levels of incorporation enhance product quality without adversely affecting sensory acceptability, highlighting the potential of spices as functional ingredients in ice cream formulation. Recommended levels reported in the literature include 0.5% turmeric combined with 0.02% black pepper powders [69]; 1.5% fenugreek, coriander, black cumin, or cinnamon powders [70]; 0.2% clove and cinnamon essential oils [73]; and 0.20% black pepper oleoresin, 0.15% ginger oleoresin, and 0.05% turmeric oleoresin [72]. Thus, in most studies, spice addition is limited to fractions of a percent. However, ice cream supplemented with nutmeg purée at a substantially higher level (9.8%) was still classified in the “like” category based on organoleptic evaluation of color, taste, aroma, and texture [74].

#### Application of Spices in Functional Meat Products

Spices such as black pepper, chili, paprika, coriander, and cumin play a key role in the preparation of meat and meat products by enhancing flavor, imparting aroma, reducing salt content, improving appearance, and protecting against oxidation and microbial spoilage, thereby extending shelf life. Modern lifestyles encourage the consumption of ready-to-eat foods, among which meat products represent a major source of dietary sodium due to the addition of salt required for flavor and safety. Excessive salt intake, however, is associated with an increased risk of hypertension and cardiovascular disease. In this context, spices offer a promising approach for reducing sodium levels in meat products while maintaining product quality and consumer acceptability [75]. In the study of Carraro et al. [76], replacing 50% of NaCl with KCl in Bologna sausage resulted in an approximately 31% reduction in sodium content without affecting emulsion stability, texture, or microbiological safety. Although the reduced-sodium formulation without spices showed lower sensory acceptance and purchase intention (36% willing to buy comparable to the control sausage with conventional salt content), the addition of herb and spice blends, % (w/w): (coriander, 0.5; onion, 0.4; white pepper, 0.1) or (cardamom 0.5; onion, 0.3; Jamaican pepper, 0.2) improved sensory quality, restoring consumer purchase intention to 52–54%.

Replacing 0.5% of NaCl (from 1.5%) in dry-cured sausage with 1.0% NaCl and 0.5% microencapsulated spices and aromatic plant extracts resulted in significantly lower lipid oxidation at the end of the storage period compared with the control, as reflected by reduced TBARS values (0.35 vs. 0.78 mg MDA/kg). This indicates enhanced oxidative stability associated with the antioxidant activity of the encapsulated plant compounds and demonstrates their effectiveness in retarding lipid oxidation during storage [77].

Application of plant materials can serve as a replacement for synthetic antioxidants used in the meat industry, meeting growing consumer demand for natural foods [78, 79]. Recent studies show that spices can effectively inhibit oxidative degradation and the formation of processing-related hazardous substances in cooked meat products. In roast beef patties, the addition of turmeric at 0.5% (w/w) significantly reduced lipid oxidation: malonaldehyde (MDA) levels decreased by approximately 48% compared to the control sample. Furthermore, turmeric significantly suppressed the formation of reactive carbonyl compounds during thermal processing, reducing glyoxal by approximately 15% and methylglyoxal by 21.3%. This highlights its strong antioxidant potential and its suitability as a natural alternative to synthetic antioxidants in thermally processed meat products [80].

Pre-soaking meat for dendeng (traditional Indonesian thinly sliced dried meat) in a spice solution containing 1% coriander and 5% garlic for 12 h reduced residual nitrite and malonaldehyde (MDA) levels, which was associated with the suppression of oxidative processes in the meat system. The spices used are rich in phenolic and sulfur-containing compounds with reducing and antioxidant activity, which accelerate the conversion of nitrite to nitric oxide and simultaneously inhibit lipid oxidation, thereby lowering MDA formation. This is considered beneficial from a consumer health perspective, as it may decrease the potential formation of carcinogenic nitrosamines and reduce dietary exposure to lipid oxidation products [81].

#### Application of Spices in Functional Confectionery

Spice incorporation into confectionery products can enhance both nutritional value and sensory quality, increasing antioxidant capacity and improving flavor, while maintaining consumer acceptability. This demonstrates the potential of spices to transform traditionally high-sugar, low-nutrient sweets into functional foods with health-promoting benefits [82].

In confectionery, spices are incorporated in the form of powders, extracts, or essential oils, and are frequently encapsulated to preserve bioactive compounds and reduce bitterness. The addition of antimicrobial and antioxidant spices to milk candy significantly enhanced both nutritional and functional properties. In the study by Faturoti and Ogidi [83], milk candy was enriched with spices (alligator pepper, clove, cinnamon, calabash nutmeg, ginger, uziza seeds, cumin, and fennel), which accounted for approximately 10% of the total mass of the candy mixture. This enrichment significantly enhanced the functional properties of the product, with DPPH antioxidant activity increasing to 65.8% compared with 18.5% in unspiced control. Spice addition also increased fiber content (6.9–8.1% vs. 3.9% in control) and mineral content (1.1–1.6% vs. 0.9% in control), promoted the growth of probiotics such as *Lactobacillus fermentum* and *Lactobacillus acidophilus*, and maintained good sensory acceptability (7.4–7.7 vs. 6.7 in control). These results demonstrate that spice incorporation can effectively improve both bioactive potential and consumer appeal in dairy-based confectionery.

Chocolate containing 0.25% cinnamon (*Cinnamomum burmanii*) essential oil received the highest sensory score for taste among panelists, while the presence of cinnamaldehyde in the oil suggests that such enrichment may also enhance the product’s health-related properties, particularly its antioxidant activity [84]. The incorporation of 8% cinnamon (*C. burmannii*) oleoresin microcapsules into dark chocolate, using an arabic gum and maltodextrin (3:1) shell material, resulted in a high overall quality score (0.8724), with phenolic content and antioxidant activity increasing by 3.85- and 7.52-fold, respectively [85].

#### Application of Spices in Functional Beverages

Pumpkin spice is a popular North American flavor associated with the autumn season and is composed of four main spices: cinnamon, ginger, nutmeg, and allspice. The Pumpkin Spice Latte has become Starbucks’ most successful seasonal beverage, with more than 424 million servings sold in the United States since its introduction in 2003, thereby contributing substantially to consumer demand for these spices [24].

Currently, very popular trend is addition of spice to coffee. The incorporation of spice-derived ingredients into coffee has been shown to enhance both sensory quality and functional properties. Thus, addition of 1% cinnamon powder into sifted Liberica coffee increased the total sensory score (9-point hedonic scale) from 7.77 to 8.42, total phenolic content from 42.27 to 47.22 mg GAE/g, total flavonoid content from 8.43 to 10.63 mg QE/g, and antioxidant activity, expressed as IC₅₀, improved from 72.12 to 12.45 mg/L. Cinnamon also enhanced the flavor profile of coffee by accentuating spicy notes [86]. Liberica coffee supplementation with temulawak (*Curcuma zanthorrhiza)*, also known as Javanese turmeric, powder at coffee-to-spice ratio 95:5 achieved the highest sensory performance, rated as “excellent” by panelists compared with “very good” for the control. The optimal 5% formulation increased total phenolic content from 42.271 to 49.146 mg GAE/g and total flavonoid content from 8.43 to 24.667 mg QE/g. It also markedly enhanced antioxidant activity, reducing the IC₅₀ value from 72.122 to 4.984 ppm [87]. Similarly, supplementation of Robusta coffee with 10% andaliman powder, a unique Indonesian spice, also known as Batak pepper or lemon pepper, resulted in higher sensory evaluation scores and increased antioxidant activity [88].

### Application of Spice Essential Oils in Functional Products

Essential oils from spices are typically obtained using traditional distillation methods, such as steam or hydrodistillation, which remain the most widely used industrial methods. In recent years, alternative extraction methods, including solvent extraction and supercritical CO₂ extraction, have been developed to improve extraction efficiency and preserve heat-labile bioactive compounds. New technologies, such as microwave and ultrasound extraction, are also being explored as environmentally friendly and time-saving alternatives [89, 90]. It should be noted that spice essential oils may be used as food flavorings only when listed as GRAS by the U.S. FDA (Food and Drug Administration) and applied strictly at low, food-grade concentrations.

#### Antimicrobial Properties of Spice Essential Oils

Essential oils are well known for their strong antimicrobial activity against various foodborne and opportunistic pathogens, such as *Escherichia coli*, *Staphylococcus aureus*, *Listeria monocytogenes* and others [91, 92]. Their antimicrobial mechanisms include disruption of cell membranes, inhibition of protein and nucleic acid synthesis, interference with ATP production, induction of oxidative stress, and direct interaction with bacterial DNA, particularly by phenolic constituents [93–95]. Antibacterial activity of popular spice essential oils is shown in Table 3.

**Table 3:** Antimicrobial activity of spice essential oils

Essential oil form spice	Active against	Reference
Black cumin (*Nigella sativa*)	MIC, µg/ml: 645 for *Escherichia coli*; 964 *for Staphylococcus aureus*; 1059 for *Klebsiella pneumoniae;* 1236 for *Enterobacter cloacae;* 815 for *Salmonella enterica*	[96]
Black pepper (*Piper nigrum*)	MIC 6.9–7.6 mg/ml for *Pseudomonas orientalis*	[97]
Cardamom (*Elettaria cardamomum*)	MIC, µg/ml: 48 for *Bacillus subtilis* ATCC 6633, *Enterococcus faecalis* ATCC 29212, *E. coli* ATCC 25922, *P. aeruginosa* ATCC 27853, *Salmonella* Typhimirium ATCC 14028, *Shigella flexenerii* ATCC 12022, *Serratia marcescens*, *Vibrio cholerae* ATCC 9459	[32]
Cinnamon (*Cinnamomum zeylanicum*)	*Paenibacillus larvae*, MIC 25–100 µg/ml; MBC 125–250 µg/ml	[98]
Chili (*Capsicum annuum*)	MIC for clinical isolates, mg/ml: 3.5 for *S. aureus*; 3.0 for *E. coli*; 3.5 for *Bacillus cereus*; 3.0 for *S.* Typhimurium	[99]
Clove (*Syzygium aromaticum*)	MIC, µg/ml: 1.25 for *E. coli* ATCC 35214, *K. pneumoniae* CIP 104727; *Lysteia monocytogenes* ATCC 19115; *P. aeruginosa* ATCC 27853; *S. aureus* ATCC 29213; *B. cereus* ATCC 14579, *Aspergillus niger* CCMM M100; 2.5 for *C. albicans* CCMM L4	[35]
Cumin (*Cuminum cyminum*)	MIC, µg/ml: 1 for *Streptococcus pyogenes;* 2 for *L. monocytogenes* and *B. subtilis*; 16 for *Enterobacter aerogenes*; 4 for *Salmonella enterica* serovar Typhimurium; 8 for *Shigella dysentery*	[36]
Fennel (*Foeniculum vulgare*) seeds	*E. coli*,* P. aeruginosa*,* Salmonella enterica*,* S. aureus*,* C. albicans*, and *Aspergillus niger* with MIC from 2.5 to 7.5 mg/ml	[37]
Ginger (*Zingiber officinale*)	MIC, mg/ml: 1.80 for *E. coli* ATCC 25922; 1.00 for *P. aeruginosa* ATCC 27858; 2.00 for *Propionibacterium acnes* ATCC 11827 and *C. albicans* ATCC 10231	[100]
Nutmeg (*Myristica fragrans*)	MIC 15 µL/mL against *S. aureus* ATCC 25923, *E. coli* ATCC 25922, *L. monocytogenes* ATCC 19111, and *S.* Typhimurium ATCC 14028	[101]
Turmeric (*Curcuma longa*)	MIC 73.7 µg/ml for *Fusarium verticillioides* Active against *C. albicans*, *B. subtilis*, *K. pneumoniae*,* B. cereus*,* Proteus vulgaris*	[102] [103]

Although Gram-negative bacteria are generally considered less susceptible to essential oils due to the presence of an outer membrane, analysis of available data (Table 3) did not reveal consistent differences in MIC values between Gram-positive and Gram-negative microorganisms. Thus, antimicrobial activity depends not only on Gram classification, but also on the chemical composition of essential oils and strain-specific factors.

#### Antioxidant Properties of Spice Essential Oils

In addition to their antimicrobial activity, spice essential oils exhibit strong antioxidant properties, making them valuable additives in a wide range of food products. These properties not only enhance the health benefits of foods but also inhibit oxidative processes, especially in meat and dairy products, thereby helping to extend their shelf life. The antioxidant activity presented in this review is primarily based on the DPPH assay, which is widely used in research. However, it should be noted that this method has limitations, including a greater sensitivity to lipophilic antioxidants and a limited ability to fully reflect the contribution of hydrophilic compounds such as phenolic acids, flavonoids, and ascorbic acid.

The antioxidant activity of essential oils is influenced by several factors, such as cultivar variability, growing conditions, the plant part used for oil extraction, extraction conditions, and the analytical method applied for antioxidant assessment. For example, the antioxidant activity, measured by IC_50_DPPH (Half-maximal Inhibitory Concentration determined by DPPH assay), of essential oils form different parts of *Piper nigrum* was 0.255 mg/ml for stems; 0.359 for leaf, and 0.486 mg/ml for seeds [104]. The antioxidant activity, IC_50_DPPH, of *Nigella sativa* essential oil obtained with supercritical fluid extraction was 1.58 mg/ml and 2.30 mg/ml after cold pressing [105]. Essential oils from *Elettaria cardamomum* extracted with tetrafluorethane showed IC_50_DPPH 63.3 mg/ml [106] and 0.681 mg/ml when methanol was used as extractor [107]. However, using the same analytical method, it is possible to make a relative assessment of the level of antioxidant activity of different oils. Antioxidant activities of different essential oils measured by the DPPH (2,2-diphenyl-1-picrylhydrazyl) radical scavenging activity assay are shown in Table 4. Lower IC₅₀ values are associated with stronger antioxidant activity.

**Table 4:** Antioxidant activities of different essential oils measured by the DPPH assay

Essential oil form spice	IC_50_, mg/ml	Reference
Black cumin (*Nigella sativa*)	4.020	[108]
Black pepper (*Piper nigrum*)	0.486	[104]
Cardamom (*Elettaria cardamomum*)	0.681	[107]
Cinnamon (*Cinnamomum zeylanicum*)	0.491	[109]
Clove (*Syzygium aromaticum*)	0.013	[110]
Cumin (*Cuminum cyminum*)	4.200	[111]
Fennel (*Foeniculum vulgare*)	3–21	[112]
Ginger (*Zingiber officinale*)	0.065	[107]
Nutmeg (*Myristica fragrans*)	5.288	[110]
Turmeric (*Curcuma longa*)	0.092	[113]
Star anise (*Illicium verum*)	9.880	[114]

The essential oils listed in Table 4 can be arranged according to the strength of their antioxidant activity in the following descending order: clove > ginger > turmeric > black pepper > cinnamon > cardamom > black cumin > nutmeg > fennel > star anise.

Antioxidant activity of essential oils is based on several mechanisms, including radical scavenging via hydrogen atom transfer (HAT), single electron transfer (SET), and metal ion chelation [38, 110, 114]. Phenolic compounds, such as thymol, carvacrol, and eugenol, are considered the major contributors to antioxidant activity because their hydroxyl groups can readily donate hydrogen atoms or electrons to stabilize free radicals through HAT- and SET-based mechanisms. In contrast, non-phenolic terpenoids generally exhibit weaker radical scavenging activity but may contribute indirectly by quenching reactive oxygen species or enhancing synergistic antioxidant effects. In addition, phenolic compounds and some terpenoids are capable of chelating transition metal ions, thereby inhibiting metal-catalyzed formation of reactive oxygen species [108, 109]. The DPPH assay primarily reflects the ability of compounds to act through hydrogen atom or electron donation, whereas other methods, such as ABTS (2,2′-azino-bis(3-ethylbenzothiazoline-6-sulfonic acid)) and FRAP (ferric reducing antioxidant power), evaluate antioxidant capacity based on different reaction mechanisms. Therefore, the use of multiple assays may provide a more comprehensive assessment of antioxidant properties.

### Spice Essential Oils in Food Products

Essential oils in food production can be used for direct addition to food products, for surface treatment of food products both directly and by inclusion in edible coatings, and as a component of packaging materials.

#### Direct Addition of Essential Oils into Food Matrices

Numerous studies have demonstrated that the addition of spice essential oils (EOs) can enhance food biosafety and extend product shelf life due to their antimicrobial properties. For example, the incorporation of 1% cinnamon (*Cinnamomum zeylanicum*) essential oil into marinades for meat and fish inhibited fungal growth during storage at 4 °C under normal atmospheric conditions, thereby extending product shelf life, whereas the marinade itself did not suppress microbial growth [115]. The addition of 0.15% coriander (*Coriandrum sativum*) essential oil to butter cakes packaged in polypropylene film and stored at 25 °C for 60 days demonstrated strong antifungal activity; however, it reduced overall sensory acceptability [56].

The antibacterial activity of essential oils has been demonstrated in studies using food products artificially contaminated with foodborne pathogens and spoilage bacteria. Ginger (*Zingiber officinale*) essential oil, applied at 0.1 mg/ml to soft cheese inoculated with *Pseudomonas aeruginosa* (8 log₁₀ CFU/ml) or *Staphylococcus aureus* (4 log₁₀ CFU/ml), reduced the *P. aeruginosa* count by 44.1% after 4 weeks of storage and completely inhibited *S. aureus* growth after 1 week storage in sterile distilled water at 4 °C [116]. Cinnamon essential oil, added at 2% to tahini sauce inoculated with *Escherichia coli* O157:H7 (~ 4.5 log₁₀ CFU/ml), reduced the *E. coli* count by 1.38, 1.79, and 2.20 log₁₀ CFU/ml after 28 days of storage at 10, 25, and 37 °C, respectively [117].

Essential oils can be incorporated into foods in their natural form; however, their direct application is limited by low water solubility, high volatility, oxidative instability, and strong sensory impact, as well as by interactions with food matrix components that reduce bioactivity. Encapsulation technology overcomes these limitations by enhancing essential oil effectiveness and reducing undesirable interactions with food constituents [118].

**Fig. 3:**
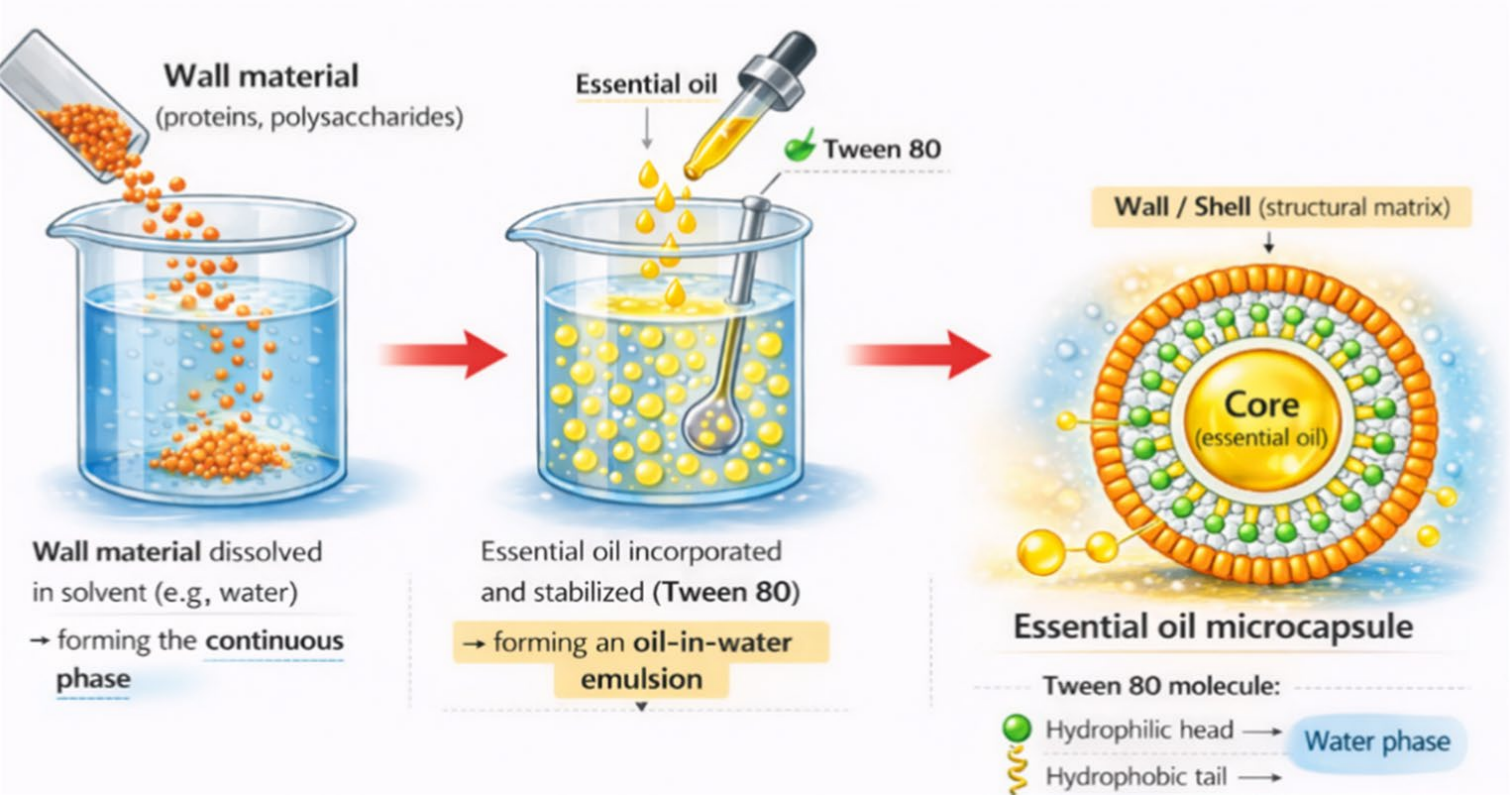
Microencapsulation of essential oil (created by the authors)

Encapsulation of essential oils (EOs) is the process of trapping oil droplets within a protective coating (shell or matrix) to reduce volatility, prevent degradation caused by light and oxygen, and enable controlled release of the active compounds. Common encapsulating agents include biopolymers and food-grade carriers such as alginate, gelatin, chitosan, maltodextrin, or gum arabic. Typically, the wall material is first dissolved in an appropriate solvent, most often water, forming the continuous phase. The essential oil is then gradually incorporated under constant stirring or homogenization to produce an oil-in-water emulsion. To improve emulsion stability and droplet dispersion, emulsifiers or stabilizers such as Tween 80, Tween 20, lecithin, or other surface-active agents may be added. These compounds reduce interfacial tension and inhibit droplet aggregation. Essential oils may be microencapsulated using methods like spray drying, coacervation, or extrusion. Subsequently, a core–shell structure is formed, in which the essential oil (core) is surrounded by a solid or semi-solid protective outer layer (shell or matrix) Fig. 3).

Encapsulation provides protection against environmental factors and allows controlled release of bioactive compounds over time. The release of encapsulated compounds is generally controlled by diffusion, matrix swelling, or degradation mechanisms and is influenced by factors such as particle size, porosity, and carrier–compound interactions, although a detailed mechanistic discussion is not the main focus of this review. Depending on particle size, encapsulation systems are commonly classified as microencapsulation (approximately 1–1000 μm) and nanoencapsulation (typically 10–300 nm). Microencapsulation primarily enhances storage stability and minimizes the loss of volatile constituents, whereas nanoencapsulation offers improved dispersion, enhanced bioavailability, and often greater antimicrobial effectiveness due to the reduced particle size and increased surface area.

Besides their antimicrobial effect, the incorporation of essential oils into food products also enhances antioxidant activity. At the same time, encapsulation allows the amount of essential oil added to be reduced while maintaining or enhancing its effect. Accordingly, clove and cumin oils were added to biscuits as non-encapsulated (5% of wheat flour), micro-encapsulated (1%), and nano-encapsulated (1%). After 60 days of storage at room temperature, clove oil in biscuits retained strong antioxidant activity, with DPPH IC₅₀ values of 2.88 µg/ml (control, without essential oil), 40.35 µg/ml (non-encapsulated), 48.22 µg/ml (micro-encapsulated), and 51.04 µg/ml (nano-encapsulated). Similarly, cumin oil showed IC₅₀ values of 4.25 µg/ml, 26.52 µg/ml, 28.54 µg/ml, and 31.48 µg/ml for control, non-, micro-, and nano-encapsulated forms, respectively. Thus, encapsulation clearly improved the antioxidant stability of both oils during storage [55].

Microencapsulated cinnamon and oregano essential oils, applied as a 1:1 mixture at 0.25% (w/w) each, reduced *Listeria monocytogenes* counts in Italian salami from approximately 10⁶ CFU/g to below the detection limit after 10 days of storage at 4 °C [119]. Addition of coriander essential oil (0.02%) to vacuum-packed minced beef stored at 6 °C for 15 days suppressed the growth of bacteria belonging to the *Enterobacteriaceae* family, showing reductions of 1–2 log cycles compared with the control sample without the additive [120].

Essential oils can also be incorporated into food products as nano-emulsions, which offer distinct technological and functional advantages. The small droplet size (< 200 nm) increases surface area, enhancing the functional properties and biological activity of essential oils. In addition, the strong physical stability of nano-emulsions (resistance to gravitational separation, flocculation, and coalescence), together with their high bioavailability and low turbidity, makes them particularly attractive for food applications [121].

In a study on labneh production, buffalo raw milk was pasteurized (85 °C, 30 min), cooled to 42 °C, and inoculated with a mixed starter culture (*Streptococcus thermophilus* and *Lactobacillus delbrueckii* subsp. *bulgaricus*). The milk was fortified with clove or ginger essential oil nano-emulsions (1 µg/ml) and stored at 4 °C for 30 days. Challenge tests with *Staphylococcus aureus* (7 log₁₀ CFU/ml) and *Aspergillus flavus* (4.1 log₁₀ CFU/ml) demonstrated significant antimicrobial effects. *S. aureus* was completely inhibited by 0.1 µg/ml nano-emulsions, while *A. flavus* counts were reduced by 100 and 35% for clove and ginger nano-emulsions, respectively. No significant changes in sensory properties or starter culture activity were observed, supporting the potential of essential oil nano-emulsions as natural preservatives in dairy products [122].

#### Spice Essential Oil in Surface Treatment of Food Products

*Surface treatment of food products* with spice essential oils can enhance safety and extend shelf life by inhibiting microbial spoilage. The most common methods for applying edible coatings to fresh product include dipping (immersing), spraying, and hand-coating [123].

Essential oils can be successfully used to protect fish and seafood from microbial spoilage [124], and cinnamon bark oil is considered one of the most effective essential oils against common foodborne pathogens [125]. Treatment with this oil helps protect fish from microbial spoilage and extend shelf life. An investigation of the effect of 1% coriander essential oil applied to the surface of fresh Atlantic salmon pieces previously inoculated with the foodborne pathogen *Salmonella enterica* serovar Enteritidis ATCC 13076 showed that, after 96 h of storage at 2 °C, the pathogen count was reduced to 3.74 log₁₀ CFU/g compared with 4.49 log₁₀ CFU/g in the control samples [126]. Spraying boar loins with black pepper essential oil (BPEO) inhibited bacterial growth in a dose-dependent manner. For *Pseudomonas* spp., initial counts of 2.05 log₁₀ CFU/g increased in the control (0% BPEO), while 0.1 and 0.5% BPEO reduced counts by 2.1 and 3.05 log₁₀, respectively, compared to the control after 9 days. Similarly, for *Enterobacteriaceae* (initially 3.1 log₁₀ CFU/g), 0.1% and 0.5% BPEO decreased counts by 1.05 and 1.85 log_10_, respectively, compared to the control at 9 days [127].

Despite their bioactivity, the poor aqueous stability and high volatility of essential oils significantly constrain their use in pure form. To address these challenges, essential oil nanoemulsions have attracted increasing attention. These systems are typically formulated as oil-in-water (O/W) emulsions with nanoscale droplets (generally 20–200 nm), enabling the effective incorporation of lipophilic bioactive compounds into aqueous media. They have been explored as edible coatings to enhance quality and extend the shelf life of fresh and minimally processed products.

Nanoemulsions are produced by reducing essential oil droplets to the nanometric scale. High-energy techniques, including ultrasonication, high-pressure homogenization, microfluidization, and high-speed stirring, employ intense mechanical forces to disrupt and refine droplets. In contrast, low-energy methods, such as spontaneous emulsification and phase inversion techniques, exploit interfacial phenomena and compositional transitions within the system to achieve nanoscale dispersion with lower external energy input.

**Fig. 4:**
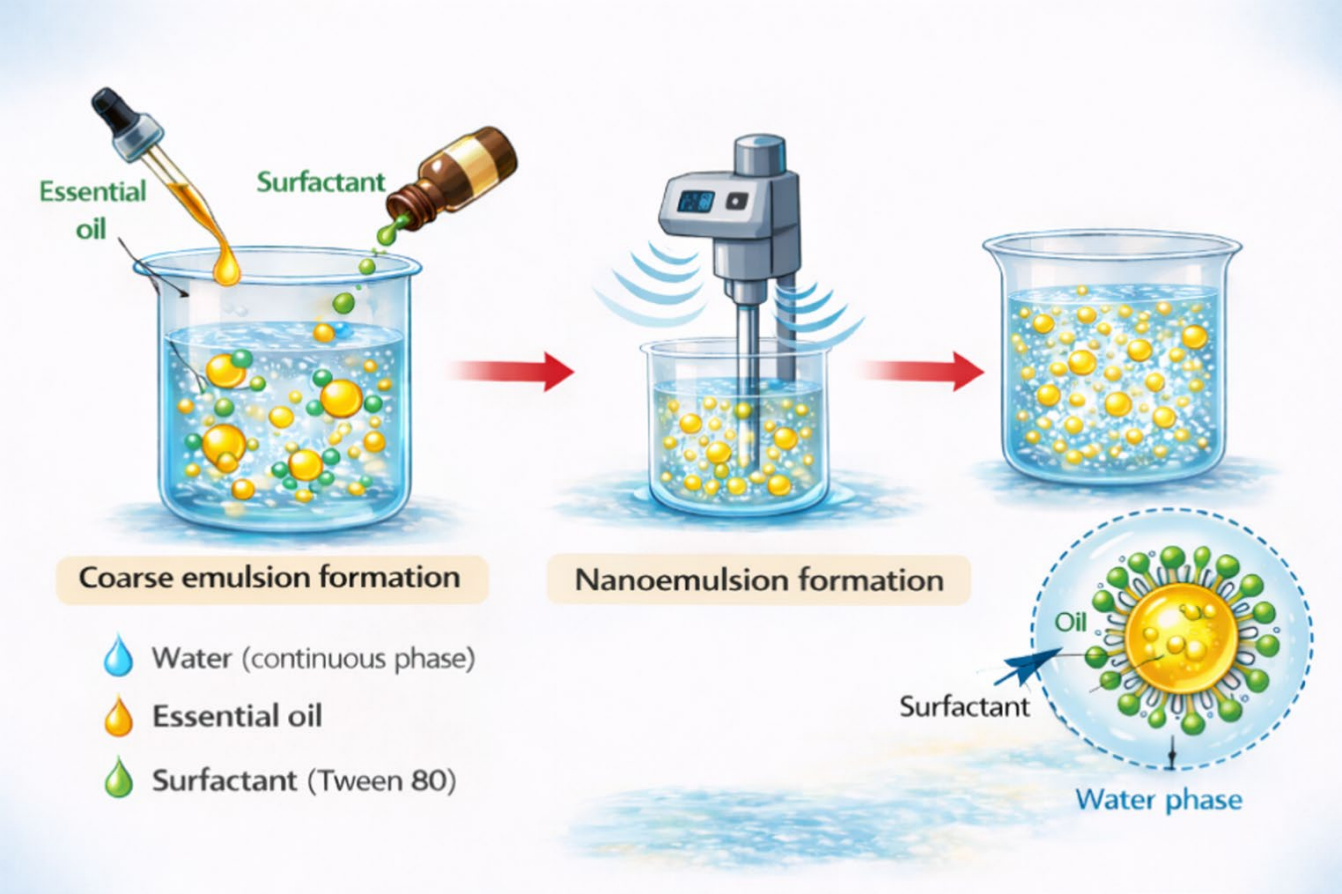
Nanoemulsification of essential oils (created by the authors)

A typical food-grade nanoemulsion consists of an oil phase, an aqueous phase, and an emulsifier. The aqueous phase is primarily composed of water, while emulsifiers such as Tween 80, lecithin, and Span 80 are commonly used to stabilize the dispersed nanodroplets by reducing interfacial tension (Fig. 4).

Grass carp fillets immersed in a cinnamon bark oil–in–water emulsion (1%, v/v) containing 0.5% food-grade surfactant (Tween 80) for 30 min at room temperature and subsequently stored at 4 °C exhibited a shelf life of 12 days based on sensory analysis, compared with 8 days for the control samples [128]. It was shown that treatment of chicken breast fillets by dipping in a nanoemulsion containing 2% (w/w) cinnamon EO for 3 min, followed by draining for 1 h, packaging in polyethylene bags, and storage at 4 °C, resulted in a decrease in comparison with untreated samples of total viable bacterial count (3.95 log₁₀ CFU/g vs. 8.50 log_10_ CFU/g) on day 15, and in *Pseudomonas deceptionensis* CM2 cells (3.30 log₁₀ CFU/g vs. 8.78 log_10_ CFU/g) on day 10 [129].

According to the FAO & WHO report [130], fresh and minimally processed fruits and vegetables are a major source of foodborne diseases. *Escherichia coli* O157:H7, *Salmonella*, and *Listeria monocytogenes* are among the most common foodborne pathogens, with documented outbreaks linked to the consumption of fresh vegetables and fruits [131–134]. Recently, increasing attention has been directed toward application on essential oils to treat fruits and vegetables, aiming to extend shelf life through the antibacterial, antifungal, and antioxidant properties of EOs [123, 135, 136]. Treatment of artificially contaminated dried honeydew melon cuts by 1-min immersion in an oil in water nanoemulsion containing 0.5% (v/v) cinnamon essential oil and 2.5% Tween 80, followed by 72 h of storage, reduced *Salmonella* spp. and *Listeria monocytogenes* counts by 3.5 and 3.0 log₁₀ CFU/g, respectively, compared with initial counts of 5.5 and 5.6 log₁₀ CFU/g [137].

*Incorporation of spice essential oils into biopolymers used to create edible coatings* is an effective way of EO application. Edible food coatings are typically composed of biopolymers such as proteins, lipids, polysaccharides, or their combinations and are applied as a thin, consumable layer on the surface of food products [138, 139]. These coatings serve as barriers against water vapor, gases, and other environmental factors, extending the shelf life of food by reducing moisture loss, regulating respiration, and preventing oxidation. The addition of spice essential oils into edible coatings enhances their antimicrobial properties, further improving food safety and prolonging shelf life of food products.

Numerous studies have confirmed the effectiveness of such edible coatings in mitigating post-harvest spoilage of fruits and vegetables. Incorporation of fennel essential oil (FEO) at 3.7% into a yam starch–based edible coating for strawberries (*Fragaria vesca* L.) inhibit mold growth, significantly enhanced the antioxidant capacity of the film, increasing the DPPH scavenging activity (IC₅₀) from 0 in the control (without FEO) to 10.05 mg film/mg DPPH. An edible coating based on yam (*Dioscorea rotundata* L.) starch containing a mixture of fennel, lavender, and lime essential oils (1.23% each), applied by spraying onto strawberries artificially inoculated with *Aspergillus niger* spores, effectively inhibited fungal growth. Complete suppression of *A. niger* was observed up to day 8 of storage at 25 °C and 85% relative humidity. Furthermore, no fungal growth was detected on non-inoculated berries coated with the essential oil mixture after 14 days of storage, whereas uncoated inoculated control samples exhibited fungal contamination in 70% of berries by day 8, and uncoated non-inoculated controls showed fungal growth in 50% of berries by day 14 [140]. These results underscore the potential of essential oil–enriched edible coatings as an effective post-harvest preservation strategy for strawberries.

Pineapple pieces, coated by immersion for 5 min in a chitosan-based edible solution (2% w/v) containing cinnamon essential oil (0.5%), packed in a polyethylene terephthalate container with a lid and stored at 5 °C, showed on day 15 compared to the uncoated control pieces lower weight loss (14.6% vs. 23.42%), higher firmness (0.30 N vs. 0.15 N), lower microbial counts (molds and yeasts: approximately 3.7 vs. 5.3 log_10_ CFU/g), and a higher overall evaluation score (3.5 vs. 2.8) [141].

Addition of cinnamon leaf essential oil (0.6%) to a chitosan-based edible coating of Braeburn apple (*Malus domestica*) slices stored at 4 °C for 9 days completely inhibited the growth of aerobic bacteria, while, total viable counts for uncoated apple slices and covered only with chitosan increased to 3.9 and 0.7 log₁₀ CFU/g on day 2, and to 5.3 and 2.0 log₁₀ CFU/g on day 9, respectively [142].

Edible coatings enriched with essential oils demonstrate significant potential for protecting meat and fish products against microbial spoilage and lipid oxidation. For example, incorporation of 1.5% cinnamon essential oil (CEO) into a chitosan-based coating (2% chitosan, 1% acetic acid, 0.75% glycerol, 0.2% Tween 80) applied to rainbow trout (*Oncorhynchus mykiss*) stored at 4 °C resulted in reduced total viable counts, lower psychrotrophic bacterial populations, and decreased thiobarbituric acid (TBA) values. After 16 days of storage, the overall sensory score of CEO-treated samples was 4.50, compared with 4.16 for samples coated only with chitosan and 1.16 for the control without coating [143].

An edible chitosan-based coating enriched with black pepper (*Piper nigrum*) essential oil proved effective for beef patties preservation, exhibiting strong repellency against carrion flies (*Calliphora vomitoria*) by preventing oviposition, maintaining its organoleptic qualities and improving colour stability during 7 days of storage. These findings underscore the coating’s potential as a natural strategy to reduce meat loss and waste, particularly under suboptimal hygienic conditions [144].

*Incorporation of essential oils in the form of nanoemulsions* into edible coatings ensures their uniform distribution, enhance their effectiveness in improving quality and prolonging the shelf life of various food products.

Application of pullulan coating (% (w/v): pullulan, 2; glycerol, 0.3; Tween 80, 0.04), incorporated with cinnamon essential oil (CEO), 0.16% (w/v) nanoemulsion, on strawberries (*Fragaria×ananassa*) reduced total aerobic bacteria and mold content (2.54 and 1.96 log_10_ CFU/g, respectively) compared to pure pullulan coating berries (3.54 and 4.66 log_10_ CFU/g respectively), on 6 day of storage at 20℃ and relative humidity 70–75% [145].

Addition of cinnamon EO nanoemulsion to a chitosan film decreased the counts of pathogenic bacteria inoculated into beef loins after chilled storage on day 21 (log₁₀ CFU/g): *Listeria monocytogenes* to 3.57 vs. 8.95; *Escherichia coli* O157:H7 was not detected vs. 2.04; *Salmonella Typhimurium* was not detected vs. 2.38 [146]. Based on the results of sensory analysis, the shelf life of beef loins was 12 days for pieces covered with chitosan only and 15 days for beef loins covered with chitosan containing cinnamon EO nanoemulsion.

### Spice Essential Oils in Active Packaging Films for Food Products

In contrast to edible coatings applied directly to food surfaces, active packaging systems incorporating essential oils rely on independent packaging materials, such as polymeric or biopolymer films, designed to intentionally interact with the food or the package headspace. Numerous studies have demonstrated that incorporating spice-derived essential oils, such as cinnamon, clove, coriander, ginger, and others, into coatings applied onto polyethylene, paper, or biodegradable substrates significantly enhances the antimicrobial and antioxidant performance of packaging systems, thereby improving their functional properties and preservation efficacy [146–154].

Biodegradable packaging films containing essential oils (EOs) therefore represent a promising strategy for active food packaging [155, 156]. Their effectiveness depends on the compatibility between the essential oils and the polymer matrix, as well as on achieving controlled release that preserves antimicrobial efficacy while minimizing undesirable sensory effects. To address challenges related to EO volatility, stability, and potential impacts on mechanical properties, microencapsulation techniques are frequently employed to enhance stability and modulate release, thereby contributing to effective and sustainable food preservation solutions.

The use of plant essential oils as natural preservatives instead of conventional chemical agents is becoming increasingly popular for prolonging the shelf life of food products [43]. Owing to their antioxidant and antimicrobial properties, essential oils have been widely investigated for incorporation into food packaging films aimed at protecting products from microbial spoilage and extending shelf life [154]. The use of natural antibacterial substances as alternatives to synthetic chemicals, which are often associated with potential health concerns, aligns with consumer preferences for safer and healthier food products. This is particularly relevant given that many essential oils used in food packaging are classified as GRAS (Generally Recognized as Safe) by the U.S. Food and Drug Administration [157]. However, once introduced into the food matrix, the volatile compounds of essential oils can be rapidly degraded by external factors such as light, temperature, and oxidation [158].

In active packaging applications, essential oils may be utilized in several ways: (a) indirect application via the package headspace, where essential oils are released from a carrier and exert antimicrobial effects in the vapor phase; (b) insertion of sachets or pads containing essential oils into the package, allowing gradual release of volatile compounds; and (c) direct incorporation of essential oils into packaging films, where they are embedded within the polymer matrix and released in a controlled manner during storage.

The direct incorporation of essential oils is more often used in the production of active packaging in the form of films that can be used as wrappers or lids, for separation of layers or simply to package food products. These films are often made from natural biopolymers such as chitosan, seaweed, rice starch, alginate, curdlan, or biodegradable synthetic polymer polyvinyl alcohol. In addition, the composition of biodegradable films includes plasticizers (more often glycerol), which improve film flexibility and prevent brittleness, and Tween 80, which acts as a non-ionic emulsifier ensuring uniform dispersion and stability of hydrophobic essential oils within the biopolymer matrix.

Incorporation of essential oils, 1–10% (w/w) into biodegradable films gives possibility to enhance preservation and prolong time of food product storage. When creating active packaging materials to enhance their antimicrobial effectiveness, special attention is paid to the use of nanotechnology [156]. Such films are extremely important for use in packaging meat products and fish. Examples of some films with spice EOs and the effect of their application on food products are presented in Table 5.

**Table 5:** Examples and effect of spice essential oil incorporation in packing film on food products during storage

Food product	Essential oil (EO), dose	Composition of film	Positive effects obtained	Reference
Beef loins	Cinnamon EO nanoemulsion,1% (v/v)	Chitosan in 1% (v/v), glycerol, 0.25 g/g chitosan	Wrapping beef pieces in a film slowed down or inhibited the growth of pathogens and allowed to extend the product’s shelf life	[146]
Pork pieces	Cinnamon EO	Polyethylene (PF) film coated with a chitosan nanoparticle emulsion containing cinnamon EO (CE-NP)	PE film coated with CE-NP suppress microbial growth and retard oxidative deterioration, extending pork shelf life	[151]
Pork belly	Clove bud EO (CBEO), 05% (w/v)	JTstarch, 2.5%, sorbitol, 1%, Tween 80, 0.05% (w/v)	Effective inhibition of lipid oxidation	[159]
Shrimps	Cinnamon EO, 2% (w/v)	Sodium alginate, 1% (w/v); glycerol (plasticizer), 0.5–1% (w/v); CaCl₂ (cross-linker), 0.07 g/g of alginate	Suppress the growth of *L. monocytogenes* during chilled storage while maintaining the sensory quality of the product	[148]
Tuna steaks	Clove EO, 0.5% (v/v)	SPI, 5% (w/v); MMT, 0.5% (w/v), glycerol, 1.25% (w/v)	Reduced microbial growth, especial *Pseudomonas* spp., and lipid oxidation	[160]
Bread slices	Cinnamon EO, 6% (w/w)	Paraffin-coated paper sheets	Suppression of *Rhizopus stolonifer* growth	[161]
Cherry tomatoes	Cinnamon EO encapsulated in starch, 0.5%	Biocomposite film from chitosan and sodium alginate	Preserving the freshness of fruit vegetables	[162]

*PVOH* Polyvinyl alcohol, *TBARS* 2-Thiobarbituric acid reactive substances, *JTS* Job’s tears (*Coix lachryma-jobi* L.) starch, *SPI* soy protein isolate, *MMT* montmorillonite clay, *CEO* clove essential oil

Application of alginate bilayer films containing cinnamon essential oil to shrimps inoculated with *L. monocytogenes* CECT 4032 (initial level 3.9 log₁₀ CFU/g) resulted in an approximately 2-log reduction in bacterial growth after 7 days of storage at 5 °C, due to the release of volatile cinnamaldehyde and eugenol, which inhibited microbial growth and slowed spoilage. In contrast, bacterial counts in shrimps covered with films without cinnamon essential oil reached 8.0 log₁₀ CFU/g [148].

Bread slices inoculated with the mold *Rhizopus stolonifer* were placed between paraffin-coated paper sheets, with 6% (w/w) cinnamon essential oil on only one side and no direct contact with the inoculated bread. Under these conditions, 96% fungal inhibition was observed after 3 days at 25 °C, indicating that the antifungal effect is due to vapor-phase transfer of volatile compounds rather than direct migration [161].

Polyethylene film coated with a chitosan nanoparticle emulsion containing cinnamon essential oil (CEO-NPs) (average particle diameter 527 nm; coating density 5 g/m²) was used to package pork samples, which were stored aerobically at 4 °C for 15 days. The initial total bacterial count (TBC) was 3.20 log₁₀ CFU/g. In control samples wrapped with uncoated film, TBC increased to 6.19 log₁₀ CFU/g by day 6, exceeding the commonly accepted microbiological limit (≈ 6.0 log₁₀ CFU/g) and restricting shelf life to 6 days. In contrast, pork packaged with CEO-NP-coated films showed pronounced inhibition of bacterial growth, with TBC reaching 6.60 log₁₀ CFU/g only on day 15, confirming the strong antimicrobial effect and extension of meat shelf life. Lipid oxidation was also reduced. The control samples exceeded the TBA (2-thiobarbituric acid) acceptability limit (2 mg malonaldehyde/kg), reaching 2.40 mg/kg on day 6, whereas treated samples showed a lower TBA value (2.09 mg/kg) after 15 days. The initial peroxide value (POV) was 1.89 meq/kg; after storage, POV increased to 4.68 meq/kg in controls but remained substantially lower (1.50 meq/kg) in CEO-NP-packaged pork, indicating effective suppression of oxidative deterioration [151].

Pork belly packaged with Job’s tears (*Coix lachryma-jobi* L.) starch (JTS) film containing 0.5% clove bud essential oil (CBEO) exhibited a lower degree of lipid oxidation during 7 days of refrigerated storage, as indicated by both peroxide value (PV) and thiobarbituric acid reactive substances (TBARS) measurements, compared with samples wrapped in JTS film without CBEO. The TBARS value, which was initially 0.36 mg MDA/kg, increased to approximately 0.58 mg MDA/kg in the control sample, whereas it remained nearly unchanged (≈ 0.38 mg MDA/kg) in the CBEO-treated sample. Similarly, the initial PV (0.24 mEq/kg) decreased to ≈ 0.12 mEq/kg in the control, while a markedly lower value (≈ 0.06 mEq/kg) was observed in the CBEO-containing film, indicating effective inhibition of lipid oxidation by the essential oil [159].

The incorporation of clove essential oil (CEO) into active nanocomposite films based on soy protein isolate (SPI) and montmorillonite clay (MMT), used to wrap bluefin tuna (*Thunnus thynnus*) steaks stored at 2 °C for 17 days, reduced microbial growth and lipid oxidation. On day 15 of storage, microbiological counts (log₁₀ CFU/g) in tuna steaks wrapped with CEO-containing films were lower than those of the control samples (films without CEO), as follows: total viable bacteria, 6.5 vs. 8.3; total aerobic mesophiles, 6.4 vs. 8.3; *Pseudomonas* spp., 6.4 vs. 7.8; *Enterobacteriaceae*, < 1 vs. 2.6. The thiobarbituric acid reactive substances (TBARS) index (mg MDA/kg product) was also reduced (0.3 vs. 0.7) [160].

The biocomposite film was composed of chitosan as the outer layer and sodium alginate as the intermediate layer. The addition of starch-encapsulated cinnamon essential oil (0.5%) to the sodium alginate layer increased the antimicrobial properties of the film, inhibiting the growth of *E. coli* and *S. aureus* by 47.2% and 75%, respectively, compared with the control film without essential oil (21.6 and 23.3%, respectively). This film was proposed for preserving the freshness of cherry tomatoes [162].

Thus, spice essential oils, owing to their antioxidant and antimicrobial properties, have considerable potential for application in active food packaging. Nevertheless, their practical use is constrained by several inherent limitations, including low solubility, high volatility, sensitivity to heat and light, and a strong aroma that may negatively affect the organoleptic characteristics of food products. Essential oils are prone to evaporation and degradation, and excessive concentrations may alter the natural flavor and aroma of foods. To improve their stability and retention within packaging materials, strategies such as nanoemulsification and encapsulation are widely employed. These approaches enhance physicochemical stability, enable controlled release, and mitigate undesirable sensory effects.

### Limiting Consumption of Spices and Their Essential Oils

Most spices have no officially established maximum intake limits. Spices are classified as Generally Recognized as Safe (GRAS) [163] by the FDA for typical culinary use, based on expert consensus and a history of safe consumption, but this does not ensure safety at excessive dosages. However, there are recommended intake limits for specific bioactive or potentially toxic components present in some spices (e.g., coumarin in cinnamon, safrole in nutmeg, capsaicin in chili peppers), and therefore risk assessments are based on analysis of individual compounds rather than on the spices themselves. As a result, recommended intake levels are typically determined based on acceptable daily intakes for individual components rather than standardized intakes for the spices themselves. For example, the acceptable intake of cinnamon is primarily defined by its coumarin content, with a tolerable daily intake of 0.1 mg/kg body weight. For an average adult, this corresponds to approximately 6–7 mg of coumarin per day, which is generally equivalent to 0.5–2 g of Ceylon cinnamon (*Cinnamomum verum*). In contrast, cassia cinnamon contains higher levels of coumarin, and regular consumption of amounts exceeding about 1 g per day may surpass the recommended limit. Additionally, allergic reactions may occur in sensitive individuals even at lower intake levels [164].

Although spices and spice-derived products provide valuable antimicrobial and antioxidant effects in food systems, their use may pose microbiological, chemical, and toxicological risks if quality control, processing, and appropriate dosages are not ensured. Therefore, proper decontamination, standardization, and regulatory compliance are essential to prevent contamination with pathogens, chemical residues, trace metals, or allergenic compounds and to protect consumer health [165].

Spices and herbs can pose an allergy risk due to the presence of pollen or plant hairs. Spices such as pepper or chili contain compounds like piperine and capsaicin, which can cause irritation, stomach upset, or discomfort in sensitive people. To reduce the risk of allergic reactions, manufacturers of spices and processed foods are required to label allergenic ingredients in accordance with EU Regulation No. 1169/2011 (the Food Information to Consumers, or FIC, Regulation). Most commercial spices now carry warnings about potential allergens or trace amounts of allergenic substances, protecting both consumers and producers.

Adulteration and mislabeling of spices may occur, including substitution of raw materials, loss of bioactive compounds, or incorrect information about their origin. For example, true Ceylon cinnamon is often replaced with cheaper cassia, which differs in aroma and bioactive properties. However, detecting such adulteration is challenging and requires advanced analytical methods [166].

Spices may be at risk of microbiological contamination and mycotoxin hazards. Pathogenic bacteria, such as *Salmonella*, are sometimes reported in chili, black pepper, and other spices, while mycotoxins, including aflatoxin B1 produced by *Aspergillus* species, a toxic metabolite that poses a significant health risk, have been detected in a number of spices, including cloves, fennel seeds, red chili, and paprika [167, 168]. Spices may also be contaminated with pesticides, plant protection products, and other agricultural chemicals introduced during crop cultivation and raw material production [169, 170]. All of these factors highlight the need for strict monitoring, quality control, and regulatory compliance to ensure spice safety.

Safety concerns also apply to the use of spice essential oils, which, due to their high concentration and biological activity, may cause toxicity, irritation, or allergic reactions if improperly used. Their safe application depends on correct dosing, purity, and adherence to regulatory standards [171]. Only essential oils derived from GRAS-listed spices should be used, and their production must rely on high-quality edible plant materials, with application at very low, carefully determined concentrations [172].

For example, wintergreen oil (methyl salicylate) is considered GRAS when used as a flavoring at trace levels, but it is highly toxic in concentrated form [173]. In addition, in active packaging systems, safety is further complicated by the potential migration of bioactive compounds into food, requiring careful evaluation of migration limits, interactions with packaging materials, and toxicological effects [174].

## Conclusions

Spices are a promising source of bioactive compounds with significant antimicrobial and antioxidant potential, relevant to modern food systems. The analysis presented in this review confirms their significant role in enhancing food safety, inhibiting microbial growth, reducing oxidative degradation, and extending the shelf life of a wide range of products. Beyond their traditional use as flavorings, spices and their essential oils are multifunctional ingredients that can contribute to the creation of environmentally friendly, high-quality food products. The use of microencapsulation, nanoencapsulation, and nanoemulsion technologies significantly improves the stability, bioavailability, and controlled release of active compounds. These technological approaches expand the practical application of spices in edible coatings, surface treatments, and active packaging systems, offering new opportunities to enhance food preservation. However, wider industrial use is limited by regulatory requirements, impact on organoleptic properties, variability in chemical composition, and challenges associated with standardization and large-scale processing. Therefore, future research should focus on optimizing formulation strategies, improving reproducibility and safety assessment, and developing cost-effective and scalable technologies for industrial application. Overall, spices and their essential oils represent a valuable resource for designing natural preservation strategies in the food industry.

## Data Availability

All data analyzed during this study are included in this published article.
